# Genetic Analysis of Genome-Scale Recombination Rate Evolution in House Mice

**DOI:** 10.1371/journal.pgen.1002116

**Published:** 2011-06-09

**Authors:** Beth L. Dumont, Bret A. Payseur

**Affiliations:** Laboratory of Genetics, University of Wisconsin, Madison, Wisconsin, United States of America; University of Chicago, United States of America

## Abstract

The rate of meiotic recombination varies markedly between species and among individuals. Classical genetic experiments demonstrated a heritable component to population variation in recombination rate, and specific sequence variants that contribute to recombination rate differences between individuals have recently been identified. Despite these advances, the genetic basis of species divergence in recombination rate remains unexplored. Using a cytological assay that allows direct *in situ* imaging of recombination events in spermatocytes, we report a large (∼30%) difference in global recombination rate between males of two closely related house mouse subspecies (*Mus musculus musculus* and *M. m. castaneus*). To characterize the genetic basis of this recombination rate divergence, we generated an F2 panel of inter-subspecific hybrid males (n = 276) from an intercross between wild-derived inbred strains CAST/EiJ (*M. m. castaneus*) and PWD/PhJ (*M. m. musculus*). We uncover considerable heritable variation for recombination rate among males from this mapping population. Much of the F2 variance for recombination rate and a substantial portion of the difference in recombination rate between the parental strains is explained by eight moderate- to large-effect quantitative trait loci, including two transgressive loci on the X chromosome. In contrast to the rapid evolution observed in males, female CAST/EiJ and PWD/PhJ animals show minimal divergence in recombination rate (∼5%). The existence of loci on the X chromosome suggests a genetic mechanism to explain this male-biased evolution. Our results provide an initial map of the genetic changes underlying subspecies differences in genome-scale recombination rate and underscore the power of the house mouse system for understanding the evolution of this trait.

## Author Summary

Homologous recombination is an indispensable feature of the mammalian meiotic program and an important mechanism for creating genetic diversity. Despite its central significance, recombination rates vary markedly between species and among individuals. Although recent studies have begun to unravel the genetic basis of recombination rate variation within populations, the genetic mechanisms of species divergence in recombination rate remain poorly characterized. In this study, we show that two closely related house mouse subspecies differ in their genomic recombination rates by ∼30%, providing an excellent model system for studying evolutionary divergence in this trait. Using quantitative genetic methods, we identify eight genomic regions that contribute to divergence in global recombination rate between these subspecies, including large effect loci and multiple loci on the X-chromosome. Our study uncovers novel genomic loci contributing to species divergence in global recombination rate and offers simple genetic explanations for rapid phenotypic divergence in this trait.

## Introduction

Meiotic recombination fulfills dual roles in genetics and evolution. In many species, including mammals, the proper segregation of homologous chromosomes at the first meiotic division is contingent on the presence of at least one well-positioned crossover per homologue pair [1]–[3]. The improper patterning of recombination events across chromosomes can lead to aneuploidy, a significant risk factor for fetal loss and developmental disability in humans [4]. In addition, recombination influences the evolutionary dynamics of populations by rearranging existing patterns of allelic variation to generate novel multi-locus genotypes. This genetic shuffling can increase the efficacy of natural selection by decoupling high fitness alleles from linked deleterious variation [5]–[7]. At the same time, recombination can facilitate the removal of deleterious variation from the gene pool [8], [9].

The amount of recombination per unit DNA (*i.e.* the rate of recombination) exhibits tremendous variation among species and between individuals. In mammals, the mean rate of recombination across species genomes varies by an order of magnitude [10]–[12]. Likewise, there is considerable heterogeneity in the global crossover rate among individual humans [13]–[17], house mice [18],[19], dogs [20], cows [21], and shrews [22]. Fine-scale recombination rates display similar trends: the genomic locations of recombination hotspots are not conserved between humans and chimpanzees [23], [24], and hotspots that segregate as presence/absence polymorphisms are common in human populations [25], [26] and among closely related laboratory strains of house mice [27]–[29].

Classical genetic experiments established that the rate of recombination is a complex genetic trait [30]–[33]. More recently, specific genes that influence genome-scale recombination rate variation in humans have been identified [17], [34], [35]. *Prdm9*, a meiosis-specific histone H3 methyltransferase, was recently found to control the genome-wide distribution of recombination hotspots in mice [36] and humans [37]–[39]. Despite these exciting advances in our understanding of *population variation* in recombination rate, genetic explanations for the large differences in recombination rate *between species* remain elusive. Fundamental questions have never been addressed experimentally: How many loci contribute to species divergence in recombination rate? What are their effect sizes and modes of inheritance? Where in the genome do species recombination rate modifiers reside? Do loci that control the positioning and activity of recombination within species also contribute to recombination rate differences between species? Answers to these questions are essential for understanding how the rate of recombination evolves.

To date, most genetic studies of recombination rate variation have measured recombination rates using patterns of marker inheritance in large human pedigrees [16], [17], [35] or in experimental crosses [29], [40], [41]. However, recombination rates are estimated from genetic data with considerable statistical uncertainty owing to the independent assortment of recombinant chromatids at meiosis [42]. In pedigree-based studies, this error is further compounded by the limited number of meiotic transmissions surveyed per individual. In addition, the inability to eliminate environmental contributors to phenotypic variation in humans adds even more noise to recombination rate estimates. These sources of error result in a marked loss of statistical power to find genomic regions contributing to recombination rate variation through linkage or association analysis.

A powerful alternative approach to the genetic dissection of recombination rate variation is to combine experimental crosses with cytological measures of recombination rate [43]. In particular, the immunolocalization pattern of the mismatch repair protein MLH1 along the mature synaptonemal complex has been shown to accurately and faithfully reproduce the distribution of meiotic crossovers in late pachytene spermatocytes [14], [44]–[46] and oocytes [47]. The MLH1 method for measuring recombination rate offers several notable advantages over traditional genetic approaches. First, because crossovers are directly observed, recombination rate estimates are not affected by binomial sampling of recombinant chromosomes at meiosis. Second, large numbers of spermatocytes or oocytes can be analyzed to yield precise estimates of the global recombination rate for single individuals. Third, though laborious, this method is inexpensive compared to the costs of genotyping many offspring from a single individual (as required by pedigree-based methods).

We use the MLH1 immunocytological method to demonstrate that males from wild-derived inbred strains of the house mouse subspecies *Mus musculus musculus* have markedly increased genome-scale recombination rates relative to the closely related subspecies *M. m. castaneus* and *M. m. domesticus*. We identify multiple genetic determinants of this substantial divergence in global recombination rate, providing an initial portrait of the genetic basis of species differences in this key genomic parameter.

## Results

### Variation in Global Recombination Rate among House Mouse Subspecies

**Figure 1:**
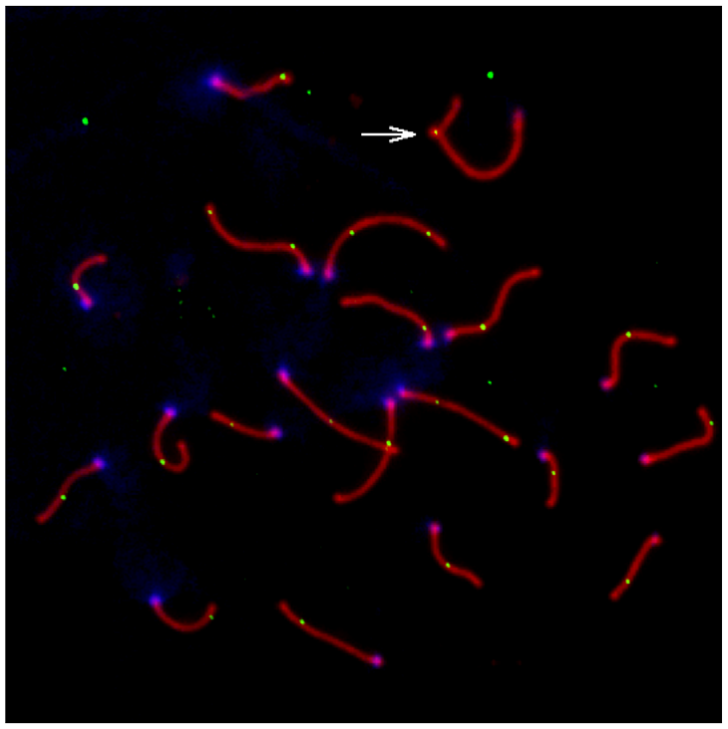
Representative pachytene spermatoctye from an inbred CAST/EiJ male. SYCP3, a component of the lateral elements of the synaptonemal complex, is stained in red. Sites of recombination along the synaptonemal complex are denoted by green MLH1 foci. Centromeric proteins targeted by CREST antibodies are in blue. The white arrow points to the heterogametic sex chromosomes. Only MLH1 foci on autosomal bivalents were scored in this study (n = 22 for this image).

**Figure 2:**
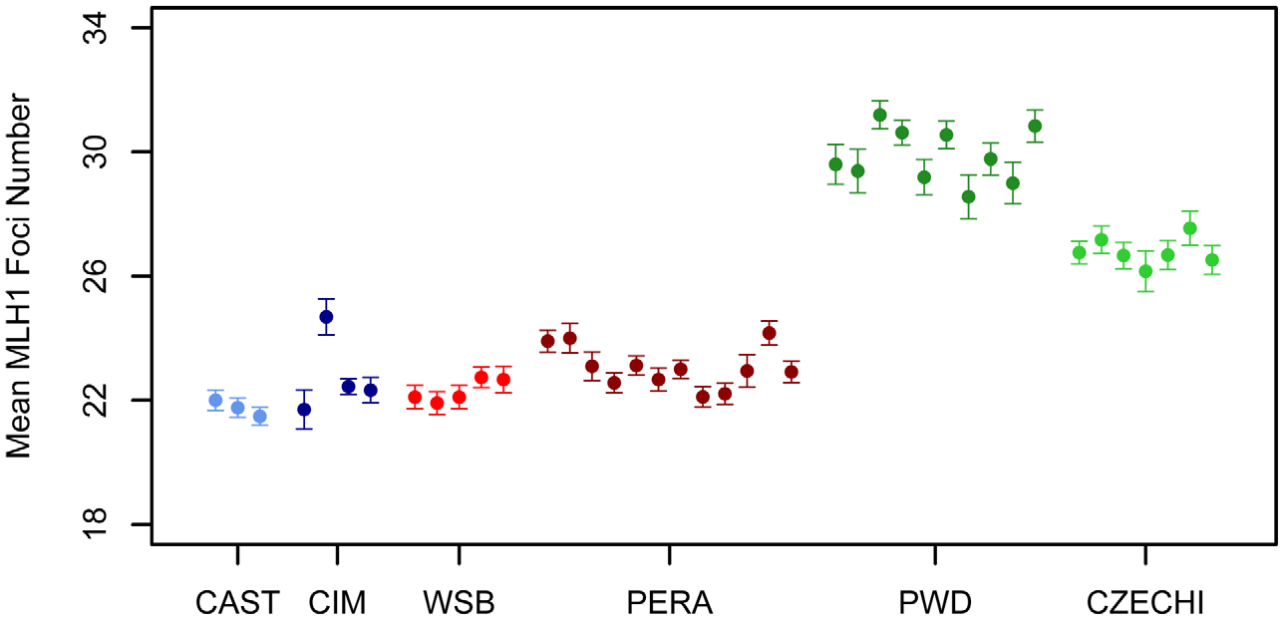
Variation in mean MLH1 foci number among *M. m. musculus*, *M. m. castaneus*, and *M. m. domesticus*. Mean MLH1 counts (±2 standard errors) were obtained from multiple males for independent wild-derived inbred strains of *M. m. musculus* (CZECHI/EiJ and PWD/PhJ), *M. m. castaneus* (CIM and CAST/EiJ), and *M. m. domesticus* (WSB/EiJ and PERA/EiJ).

A representative image of a late pachytene spermatocyte stained with fluorescently labeled antibodies against MLH1 and a protein component of the synaptonemal complex (SYCP3) is shown in Figure 1. We used the MLH1 immunostaining assay to measure genomic recombination rates in two wild-derived inbred strains from each of three distinct subspecies of house mice (*Mus musculus musculus, M. m. castaneus, and M. m. domesticus*) [48]. We observed a striking difference in mean MLH1 foci count between *M. m. musculus* and both *M. m. domesticus* and *M. m. castaneus* males (Figure 2; Wilcoxon signed rank test, *P*<10^−16^). On average, *M. m. castaneus* and *M. m. domesticus* have 21–23 MLH foci per meiosis, whereas *M. m. musculus* males undergo >26 crossovers.

Several patterns suggest that the distribution of MLH1 foci along the synaptonemal complex (SC) faithfully mirrors the distribution of meiotic crossovers in the wild-derived inbred strains we examined. First, SCs lacking a MLH1 focus were rare in our survey (0.4%), consistent with the obligate chiasma requirement for homologue disjunction in mammals [1], [2]. Second, on SCs with two or more MLH1 foci, foci were distantly spaced. This patterning matches expectations under a model of positive crossover interference, a process that is known to be important in house mice [42]. Third, we very seldomly observed cells with two or more SCs lacking a MLH1 focus. Pachytene spermatocytes nearly always had a full complement of foci, indicating that MLH1 protein loads on and off sites of recombination repair along the SC in a highly concerted fashion. Fourth, our cytology maps approximate the total male mouse genetic map length estimated from genetic data. Assuming that each MLH1 focus corresponds to a map distance of 50 cM, wild-derived inbred strains included in our survey have map lengths that range from 1085 cM–1500 cM. This range includes the estimate of total male genetic map length from the standard mouse map (1375 cM) [49]. Although the small fraction of crossover events that are not dependent on MLH1 will be missed by this method [50], our observations suggest that the total number of MLH1 foci in a meiotically dividing cell provides a reliable estimate of the genomic recombination rate.

*Mus musculus* subspecies radiated nearly simultaneously from a common ancestor ∼500,000 years ago [51], [52]. The striking increase in mean MLH1 foci count in inbred strains of *M. m. musculus* relative to the *M. m. domesticus* and *M. m. castaneus* strains suggests that considerable divergence in male recombination rate has accrued along the *M. m. musculus* lineage. Under a neutral model of phenotypic evolution, the expected recombination rate divergence between subspecies is ≈*2V_m_t*, where *V_m_* is the per-generation rate at which phenotypic variance increases via neutral mutation and *t* is the divergence time in generations [53]. Given that *t* is roughly equal for pairwise comparisons between house mouse subspecies and assuming constancy of *V_m_* over this short evolutionary period, absolute divergence in recombination rate should be approximately equal among subspecies pairs. Clearly, our data are not consistent with this theoretical prediction. At mutation-drift equilibrium, the amount of within subspecies polymorphism for recombination rate is ≈*2N_e_V_m_*, where *N_e_* is the effective population size [53]. Curiously, *M. m. musculus* has a smaller estimated *N_e_* than either *M. m. domesticus* or *M. m. castaneus* (∼60,000, 100,000, and 200,000, respectively; [52]) yet displays the highest level of polymorphism for recombination rate (∼3 MLH1 foci between CZECHI and PWD). The higher polymorphism for recombination rate in *M. m. musculus* and greater divergence for recombination rate in comparisons with this subspecies are consistent with several evolutionary hypotheses. Recombination rates may have experienced a relaxation of selective constraint along the *M. m. musculus* lineage. Alternatively, recombination rates in *M. m. domesticus* and *M. m. castaneus* may have been subject to stronger purifying selection. These findings could also be explained by higher mutational variance for recombination rate in *M. m. musculus*. We caution that these observations derive from comparisons of just two wild-derived inbred strains per subspecies. A detailed survey of polymorphism and divergence in recombination rate in natural populations of these three subspecies will be required to determine the underlying evolutionary processes at work.

### Genetic Differences in Global Recombination Rate between House Mouse Subspecies

**Figure 3:**
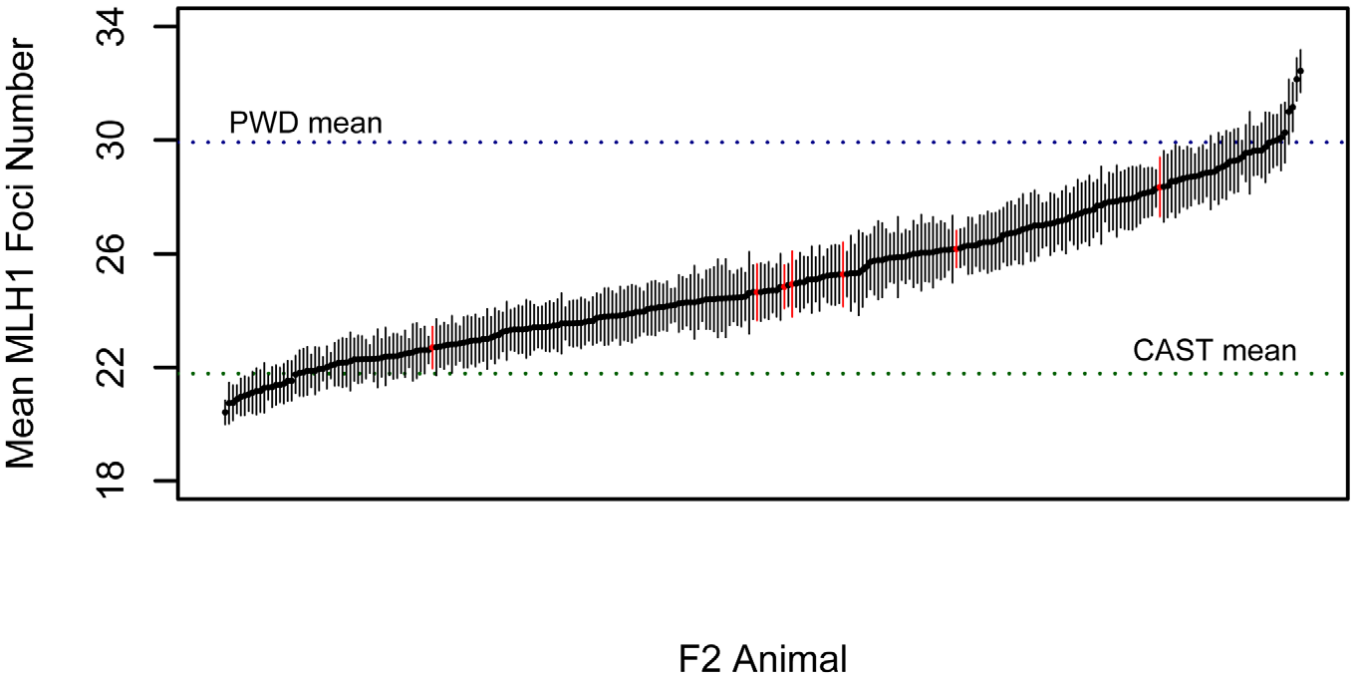
Variation in MLH1 foci count among male PWD/PhJ and CAST/EiJ F2 hybrids. Mean MLH1 counts (±2 standard errors) are shown for 269 CAST/PWD×CAST/PWD F2 males (black points and error bars) and 7 PWD/CAST×PWD/CAST F2 males (red points and error bars). Positions of the parental mean values along this distribution are shown by dashed horizontal lines.

To investigate the genetic basis of the rapid divergence in genomic recombination rate in *M. m. musuclus*, we conducted an F2 intercross between wild-derived inbred strains PWD/PhJ (*M. m. musculus*) and CAST/EiJ (*M. m. castaneus*). We measured the global rate of recombination in 276 F2 males by averaging total autosomal MLH1 foci counts from at least 15 spermatocytes per animal (mean = 20.4 cells). F2 mice vary substantially in the global number of crossovers (Figure 3). Importantly, the variation in MLH1 foci number among cells from a single male is far less than the variation in mean MLH1 foci count among animals, indicating the presence of segregating genetic differences in this inter-subspecific F2 population. Most males have recombination rates that fall within the range defined by the two parental means, although 9% of individuals lie outside these values. The continuous nature of this variation provides evidence for multiple recombination rate modifiers segregating between the parental PWD and CAST strains. The high broad-sense heritability of recombination rate in this cross (*H^2^* = 0.93) motivates the application of genetic mapping approaches to link variation in recombination rate with genetic variation at specific genomic loci.

### Single Quantitative Trait Locus (QTL) Scan for Modifiers of Genomic Recombination Rate

**Figure 4:**
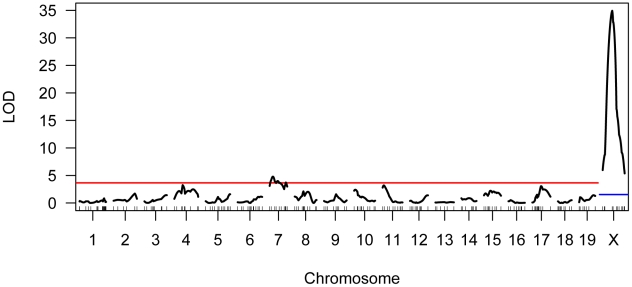
Results of single QTL mapping for F2 variation in mean MLH1 foci count. The LOD curve for each autosome and the X chromosome is displayed. The horizontal red (blue) line corresponds to the autosomal (X chromosome) significance threshold obtained by permutation with α = 0.05. Positions of genotyped markers are indicated by ticks along the x-axis.

We genotyped our F2 population at 222 informative SNPs distributed across the genome. Using standard interval mapping [54] with a permutation-derived threshold for declaring statistical significance (genome-wide α = 0.05) [55], we identified two genomic regions linked to variation in mean MLH1 foci count. One of these QTL localizes to the proximal half of chromosome 7 and the second QTL lies on the X chromosome (Figure 4). Although there is a clear peak in the X chromosome LOD profile centered on ∼30 cM, the entire chromosome displays strong statistical evidence for linkage to variation in global recombination rate (Figure 4). QTL genotype at this single, large-effect locus explains 46% of the variance in mean MLH1 foci count among F2 males (adjusted R^2^ = 0.46 from a linear regression).

Interestingly, the allele from the low recombination rate CAST parent confers the increase in recombination rate at this X-linked locus, opposite the pattern seen at the chromosome 7 QTL (Table 1). Consistent with this result, we uncover a striking difference in genomic recombination rate between reciprocal F1 males (Figure 6). Male F1 animals that receive their X chromosome from a CAST mother (CASTxPWD F1s) have ∼5 more MLH1 foci per meiosis than F1 males carrying the PWD X chromosome (PWDxCAST F1s).

**Table 1:** Estimated locations and effect sizes of QTL.

Chr	Position	∼95% CI (±1.5 LOD units)	Mean MLH1 Foci Count					Percent Variance Explained*
			CAST/CAST	CAST/PWD	PWD/PWD	CAST/Y	PWD/Y	
**Single QTL Mapping**								
7	11.0 cM18 Mb	0.5–41 cM10–97 Mb	24.18	25.03	26.00	—	—	6.70
X	31.9 cM78 Mb	14–45 cM65.5–84.5 Mb	—	—	—	26.82	23.38	45.9
**Multiple QTL Mapping**								
3	66.0 cM150 Mb	57–75 cM133–168 Mb	24.66	25.07	25.58	—	—	3.43
4	25.0 cM65 Mb	16–31.5 cM48.5–77 Mb	24.41	25.21	25.68	—	—	2.81
7	9.0 cM28.5	2–14.5 cM13.5–40 Mb	24.18	25.03	25.98	—	—	7.94
11	3.0 cM13 Mb	0–11 cM0–25.5 Mb	24.20	25.11	25.83	—	—	2.11
15	31.0 cM66.5 Mb	22–52 cM50–105 Mb	24.56	24.95	25.83	—	—	3.27
17	26.0 cM45.5 Mb	20–35 cM36.5–59 Mb	24.16	25.20	25.77	—	—	2.27
X	33.0 cM80 Mb	27.5–34.5 cM69–83.5 Mb	—	—	—	26.82	23.38	35.56
X	70.0 cM159 Mb	63.5–74 cM145–167 Mb	—	—	—	25.85	24.21	2.09

*For single QTL mapping analysis, the percent variance explained is the adjusted R2 value from a linear regression of phenotype on QTL genotype. For multiple QTL mapping analysis, estimated effects are conditioned on the presence of other QTL in the genome.

### A Multiple QTL Map of F2 Variation in Mean MLH1 Foci Count

**Figure 5:**
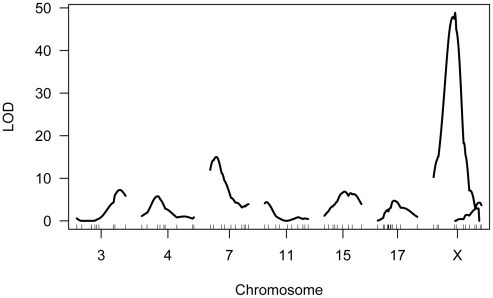
Multiple QTL map of F2 variation in mean MLH1 foci count. The subset of chromosomes with significant QTL is shown, with the positions of genotyped markers denoted by ticks along the x-axis.

Single QTL mapping approaches, including standard interval mapping, formally assume that only one QTL in the genome affects the phenotype. When QTL of moderate to large effect exist, accounting for the phenotypic variance they explain can enhance statistical power to find additional loci. The discovery of the major effect QTL on the X chromosome prompted us to use an approach that could adjust for the presence of this locus to enable the simultaneous detection of multiple additional QTL. We applied a model-based multiple QTL mapping strategy [56] to identify the set of genetic loci that best explain segregating variation in mean MLH1 foci count among F2 males. Using a forward/backward model selection approach, with model discrimination performed via penalized LOD scores to control the rate of false inclusion [57], we identify six autosomal QTL and two X-linked QTL for genomic recombination rate (Figure 5). As expected, the two QTL identified in the single QTL scan are among those recovered in the multiple QTL mapping analysis.

The six autosomal loci show mostly additive effects, with CAST alleles at the chromosome 4, 11, and 17 QTL exhibiting slight dominance over PWD alleles (Table 1). At each autosomal locus, the high recombination rate PWD parent confers the high recombination rate allele. Consistent with single QTL scan results and reciprocal F1 phenotypes, the high recombination rate allele at both X-linked QTL derives from the low recombination rate CAST parent (Table 1).

Combined, these eight QTL explain 74% of the phenotypic variance among F2s, individually accounting for 0.9–3.4 MLH1 foci (2–35% of the total phenotypic variance; Table 1). These effect sizes are probably overestimated, as the conflation of QTL detection and estimation on a common dataset leads to a systematic upward bias [58].

Although these eight QTL explain a large fraction of F2 variation in global recombination rate, they account for a lesser percentage of the observed difference between the parental PWD and CAST strains. Combined, the six autosomal QTL explain a difference of 8.5 MLH1 foci between the inbred parents – more than the observed difference of 8 foci. However, the two X-linked QTL account for ∼4 MLH1 foci in the opposite direction. Summing these effects suggests that our multiple QTL model explains approximately half of the difference in mean MLH1 foci count between PWD and CAST males. Clearly, additional QTL for mean MLH1 foci number segregate between these strains. These undetected QTL likely have small to moderate effect sizes, as power calculations indicate that our study is only sufficiently powered (80% power) to find QTL with additive effects >0.9 [59].

### Sex-Specific Evolution in Mean MLH1 Foci Count

The early stages of female meiosis, including recombination, occur in the fetal ovary. These temporal aspects of oogenesis complicate cytological analysis of recombination in females. For this reason, we limited our genetic mapping efforts to males. However, the genome-wide rate of recombination is a sexually dimorphic trait in many mammals, including house mice. The female standard mouse genetic linkage map is 9% longer than the corresponding male map [49], and marked sex-specific recombination trends are observable on finer physical scales of measurement [29], [49], [60], [61]. The non-random concentration of QTL with transgressive effects to the X chromosome, coupled with the noted sex differences in this trait, led us to investigate variation in global recombination rate in females from the two parental inbred strains and hybrid F1s.

**Figure 6:**
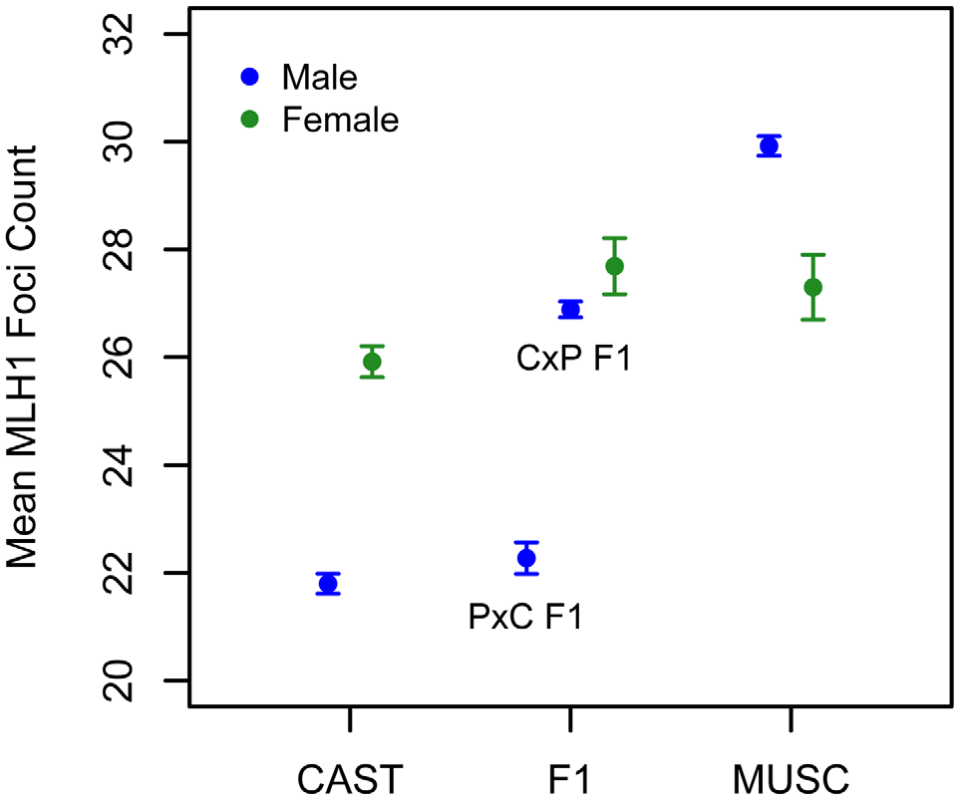
Variation in mean MLH1 foci counts (±2 standard errors) between males (blue) and females (green) of inbred CAST, PWD, and inter-subspecific CASTxPWD (CxP) and PWDxCAST (PxC) F1 origin. Strain means were calculated by pooling MLH1 foci counts over multiple animals (CAST females: data from [62]; PWD females: 3 animals; CASTxPWD F1 females: 2 animals; PWD males: 10 animals; CAST males: 3 animals; PWD×CAST F1 males: 1 animal; CAST×PWD F1 males: 11 animals).

We applied the MLH1 immunostaining procedure to oocytes harvested from day 17–20 post-conception PWD and CASTxPWD F1 female fetuses (n = 3 and n = 2 animals, respectively). Mean MLH1 foci counts from CAST females have been reported previously [62]. Although CAST and PWD males differ in their global crossover count by 8 MLH1 foci, PWD females have only 2 foci more than CAST females (Figure 6). PWD and CASTxPWD F1 females have indistinguishable crossover counts. Overall, there is surprisingly little variation for mean MLH1 foci count among females, indicating that evolutionary divergence in recombination rate has occurred primarily in males. These findings suggest that several of the QTL detected in our inter-subspecific F2 male population may be sex-limited in their expression or have polarizing effects in males versus females [17].

Interestingly, PWD females have a lower mean MLH1 foci count than PWD males (Figure 6). This finding presents an intriguing directional reversal of the global recombination rate sex dimorphism widely observed in house mice [49], [61], nominating the PWD strain as an excellent model for understanding the causes of sex differences in this phenotype.

## Discussion

### The Genetic Architecture of Recombination Rate Divergence in Male House Mice

Our genetic study of variation in mean MLH1 foci number in an inter-subspecific panel of F2 males identifies QTL for global recombination rate divergence. Our findings provide initial clues toward the genetic mechanisms of species divergence in this trait.

First, the discovery of eight QTL jointly explaining 74% of the variance among inter-subspecific F2 males indicates that observed patterns of recombination rate evolution are dominated by loci with large phenotypic effects. The X-linked QTL at 33 cM is the strongest modifier of global recombination rate identified to date, explaining 35% of the variation in our inter-subspecific F2 panel (Table 1). The presence of such a large effect locus provides a clear genetic mechanism for rapid phenotypic evolution between species.

Second, the autosomal loci we identify display mainly additive inheritance. This finding extends studies of within species recombination rate variation in humans [34], indicating that additive alleles contribute to both within and between species differences in recombination rate.

Third, at least two of the QTL we identify exert *trans* effects on recombination rate. Males do not recombine along their X chromosome, indicating that the two X-linked QTL act strictly in *trans*. This result corroborates findings from genetic studies of fine-scale recombination rate control: *Prdm9* regulates recombination hotspot activity across the mouse genome [36], indicating that *trans* regulatory mechanisms are important for both the fine- and broad-scale control of recombination.

Fourth, our multiple QTL map points to the presence of high and low recombination rate alleles in the two parental strains (Table 1). A similar pattern has been previously reported for recombination rate variation in *Drosophila melanogaster* [32] and is often observed in the evolution of complex traits [63].

Finally, our study uncovers a prominent role for the X chromosome in the evolution of recombination rate. Combined, the two X-linked loci in our multiple QTL model account for a difference of 4 MLH1 foci (200 cM) between males hemizygous for CAST versus PWD alleles. Recessive X-linked loci subject to positive selection will reach fixation more rapidly than autosomal loci because their expression is unmasked in hemizygous males [64]. We speculate that selection on the X-linked modifiers identified in our F2 male population may have played a leading role in the rapid evolution of recombination rate in this sex.

Recently, Murdoch *et al.* [43] used the approach applied in our study – genetic mapping of MLH1 foci count in F2 males – to identify seven QTL conferring recombination rate differences between the C57BL6 and CAST inbred mouse strains. Five of these loci map to chromosomes that harbor QTL in our study, including the X chromosome (chromosomes 3, 4, 15, 17, and X). Interestingly, the CAST genotype at the X-linked QTL was associated with only a moderate increase in F2 recombination rate in this study, as opposed to the large effect observed in our cross (males with the CAST X have ∼1 focus more than males with the C57BL6 X chromosome; in comparison, males with the CAST X have ∼2.5 foci more than males with the PWD X chromosome). If the large-effect X-linked QTL at 33 cM identified here and the X-linked QTL identified by Murdoch *et al.* [43] are the same locus, it would appear that genetic background strongly affects its expression. Our application of multiple QTL mapping did not identify any genetic interactions (even when the penalty to QTL inclusion was relaxed; data not shown), but we acknowledge limited power to find interacting QTL with our small sample size.

### No Effect of Inter-Subspecific Hybridization on Recombination

Allelic incompatibilities that decrease reproductive fitness in hybrids commonly evolve between incipient species [65]–[67]. These genetic incompatibilities often affect hybrid fitness by hindering progression through meiosis, including impairment of chromosome synapsis and recombination [e.g. 68]. Importantly, we detect no epistasic interactions between subspecies-specific alleles in our cross. Several additional observations indicate that observed F2 variation in mean MLH1 foci count is not due to hybridization-related defects in meiosis. First, our immunostaining assay allowed us to identify diplotene stage cells in all F1 and F2 animals, ruling out wide-spread activation of the pachytene meiotic checkpoint as an underlying mechanism of possible hybrid sterility [69]. Second, we observed no overt defects in chromosome pairing or synapsis in any hybrid animals. If CAST and PWD hybrids suffer fitness reductions, the molecular mechanism(s) of infertility must act after the completion of recombination at prophase I. It is difficult to imagine how any problems that surface late in meiosis (or possibly in spermatogenesis) could affect recombination. Third, the distribution of mean MLH1 foci counts in our F2 population is centered on the mid-parent mean (Figure 3), an unlikely result in the event of hybrid dysgenesis in the phenotype. Finally, we note that *M. m. musculus* and *M. m. castaneus* hybridize in nature [70] and F1s from both directions of our CAST and PWD cross were fertile. Together, these observations provide a compelling case that patterns of inter-subspecific hybrid variation in mean MLH1 foci count reflect underlying subspecies differences in the rate of recombination *per se*.

### Sex-Specific Divergence in Recombination Rate

Our analysis uncovered two striking differences in recombination rate evolution between the sexes. First, the magnitude of evolutionary change is much greater in males than females. Second, there is a reversal in the direction of the sex-dimorphism for recombination rate between PWD and CAST. It is tempting to consider these results, combined with the localization of both transgressive QTL to the X chromosome, as inter-related findings. In particular, they seem to raise the possibility that divergence in recombination rate among house mouse subspecies has been shaped by conflicting evolutionary pressures on the sexes. If natural selection favors distinct recombination rates in males and females [71], [72], modifiers might preferentially aggregate on the X chromosome [73]. Recessive X-linked loci will differ in expression between males and females, thereby imposing a reduced fitness burden on the opposite sex. This scenario is speculative, especially given that the dominance effects of X-linked recombination rate modifiers identified here cannot be determined from hemizygous F2 males. An extension of our QTL analysis to include mean MLH1 foci counts in F2 females will offer further clues into the evolution and genetic basis of these intriguing observations.

Although the rate of recombination differs between males and females of many mammalian species [13], [16], [19], [74], [75], the causes of this pattern remain poorly understood. Sex differences in crossover interference [76], features of the meiotic cell cycle [77], [78], the strength of epistatic selection in haploid gametes [72], and the genetic architecture of recombination [17], [35] may play contributing roles. Few X-linked recombination rate modifiers have been previously identified [43], [79], but our recent findings suggest that sex-linked loci are pervasive components of the genetic architecture of recombination rate evolution in house mice [this study]; [ 48,80]. Further examination of the genetic basis of recombination rate should allow the relative importance of sex chromosome evolution and other causes of sexual dimorphism to be determined.

### The Identification of Genes Contributing to Divergence in Recombination Rate

*Prdm9* is the only gene known to contribute to species differences in recombination rate. Human and chimpanzee alleles of *Prdm9* are predicted to recognize and bind distinct DNA sequence motifs that may be important for recombination hotspot initiation [37], [81], [82]. These observations have led to the hypothesis that rapid evolution at *Prdm9* underlies abrupt shifts in the distribution of recombination hotspots between species [37], [81]. Although the CAST and PWD strains used in our study have different functional variants of *Prdm9* [36], we do not find QTL that co-localize with this gene. *Prdm9* modifies the activity of multiple hotspots in mice [36] and in humans [38], but it does not appear to have detectable effects on the global level of recombination in either species [this study; 37]. Taken together, these findings suggest that recombination rate evolution on fine and broad scales could be controlled by separate genes [83].

While examining the genetic control of hotspot activity can deliver mechanistic insights into recombination rate evolution, the total number of recombination events in a meiotically dividing cell – the phenotype examined here – is more likely to be a functionally relevant measure [84]. For example, female reproductive output is associated with global recombination rate in humans [15], [16], whereas the presence or absence of recombination activity in individual hotspots has yet to be linked to variation in fitness. In fact, the rapid evolutionary turnover of recombination hotspots within [25], [26], [39], [85], [86] and between species [23], [24] seems to argue against a selective advantage of particular hotspot locations over others. In contrast, the genomic rate of recombination is subject to evolutionary constraints imposed by its essential functions in mammalian meiosis. A minimum of one crossover per chromosome is required for the proper disjunction of homologs in mice whereas high recombination rates may elevate the frequency of deleterious rearrangements [87].

Our study nominates eight genomic regions contributing to evolutionary divergence in genomic recombination rate. Future work will be required to determine whether the causal alleles are fixed or shared between *M. m. musculus* and *M. m. castaneus*. The observation that independent wild-derived inbred strains of *M. m. musculus* and *M. m. castaneus* conform to the recombination pattern established by PWD and CAST (Figure 2) suggests that at least some of these QTL represent subspecies differences.

The QTL identified in our analysis have broad peaks, each spanning a genomic interval that includes hundreds of genes. As a first step toward the identification of the causal variant(s) underneath the large X-linked QTL at 33 cM, we assayed transcript abundance between PWD and CAST alleles at 12 candidate genes. We found a suggestive difference in allele-specific expression at one gene, *Brcc3*, a component of the BRCA1-BRCA2 complex involved in double-strand break repair (Figure S1; Text S1; Table S1) [88]. These considerations nominate *Brcc3* as a putative candidate gene for divergence in recombination rate. However, fine-mapping strategies will be required to test this hypothesis and to further localize the genetic changes that contribute to the increased global recombination rate in PWD.

Genetic and ecological resources will facilitate the fine-mapping of QTL identified in our experimental intercross. The Collaborative Cross, an eight-way recombinant inbred line panel currently under development, includes inbred strains CAST and PWK [89], a close relative of PWD. The increased mapping resolution and ability to measure mean MLH1 foci count on multiple animals with identical genotypes are key advantages of this resource that will aid efforts to fine-map those QTL that are common between PWK and PWD. In addition, populations of *M. m. musculus* and *M. m. castaneus* hybridize in nature, forming a fourth widely recognized subspecies of house mouse, *M. m. molossinus* [70]. The genetic properties of these natural hybrid populations are not well characterized, but lower levels of linkage disequilibrium could allow genomic windows containing causative loci to be narrowed through association studies or admixture mapping [90]. Identifying the determinants of the marked divergence in male recombination rate between *M. m. musculus* and *M. m. castaneus* promises to reveal the mechanisms of sex-limited evolution in this important phenotype.

## Methods

### Animal Husbandry and Ethics Statement

Wild-derived inbred strains of *Mus musculus castaneus* (CAST/EiJ) and *Mus musculus musculus* (PWD/PhJ and CZECHI/EiJ) were purchased from the Jackson Laboratory (Bar Harbor, Maine, USA) and housed in the University of Wisconsin School of Medicine and Public Health mouse facility according to animal care protocols approved by the University of Wisconsin Animal Care and Use Committee. Pups were weaned into same-sex groupings at 21 days, with males subsequently separated into individual cages prior to 56 days. Animals were provided with food and water *ad libitum*. A total of 315 F2 males were sacrificed at 70 (±3) days of age (305 CAST/EiJ×PWD/PhJ and 10 PWD/PhJ×CAST/EiJ).

Males from inbred strain CIM were purchased from Dr. Francois Bonhomme's stock repository at the Universite Montpellier II. Animals were sacrificed shortly after arrival to the University of Wisconsin-Madison (aged 24.5–35.5 weeks).

### Meiocyte Spreads

Spermatocyte spreads were prepared as described [91]. Briefly, the left testis of sexually mature males was removed, weighed, and rinsed in sterile 1× PBS. The outer tissue coating of the testis was punctured to allow a small volume of seminiferous tubules to be extracted. Tubules were incubated in a hypotonic solution (30 mM Tris, 50 mM sucrose, 17 mM citric acid, 5 mM EDTA, 2.5 mM dithiothreitol, 0.5 mM phenylmethanesulfonylfluoride) for approximately 45 minutes at room temperature. Tubules were then transferred to a small volume (20 µl) of 100 mM sucrose solution deposited on a clean glass slide and shredded using fine-gauge forceps. Tubular remnants were removed and an additional 20 µl of 100 mM sucrose added to the cell slurry. The solution was agitated by pipetting and 20 µl deposited onto each of 2 3×1″ glass slides coated with 100 µl 1% paraformaldehyde supplemented with TritonX-100 (0.15%; pH = 9.2). The slides were gently rocked to distribute cells across their surface and allowed to dry overnight in a room temperature humid chamber. Dried slides were then washed briefly in 0.4% PhotoFlo (Kodak), air dried, and subjected to immunostaining.

### Immunostaining

The immunostaining protocol was adapted from Anderson *et al.* [45] and Koehler *et al.* [46]. A 10× concentration of antibody dilution buffer (ADB) was prepared (2.5 mL normal donkey serum (Jackson ImmunoResearch), 22.5 mL 1× PBS, 0.75 g bovine serum albumin (Fraction V; Fisher Scientific), and 12.5 µl TritonX-100) and sterilized by vacuum filtration (0.45 µm; Millipore). Slides were blocked in 1× ADB (diluted in 1× PBS) for approximately 30 minutes then lightly drained by touching the edge of the slide to a clean paper towel. All antibody dilutions were made into 1× ADB and all incubations were performed in a 37 C humid chamber. A 60 µl aliquot of primary antibody cocktail (1∶50 rabbit polyclonal antibody against MLH1 (Calbiochem) and 1∶50 goat polyclonal antibody against SYCP3 (SantaCruz Biotechnology)) was dispensed on each slide. Slides were cover-slipped, sealed with rubber cement, and incubated overnight. The rubber cement was then carefully removed and coverslips were soaked off in 1× ADB. Slides were washed twice for 30 minutes each in 1× ADB. A 60 µl volume of 1∶100 Alexa 488 donkey anti-rabbit secondary antibody (Molecular Probes) was deposited on each slide. Slides were cover-slipped, sealed with rubber cement, and incubated overnight. After soaking off coverslips in 1× ADB, 60 µl of 1∶100 Alexa 568 donkey anti-goat secondary antibody (Molecular Probes) was applied to each slide. Slides were sealed with a parafilm “coverslip” and incubated for 2 hours. Slides were then washed three times for one hour each in 1× PBS, air-dried, and mounted in a drop of ProLong Gold antifade media (Molecular Probes).

### Imaging and Pachytene Cell Scoring

Cells were visualized using a Zeiss Axioskop microscope equipped with an AxioCam HRc camera and a 100× objective lens. Late pachytene cells that were damaged during preparation, displayed bulbous chromosome termini (indicative of transition into diplotene), lacked clear cell boundaries, or displayed flagrant defects in synapsis were not imaged. Images were captured in AxioVision (Rel. 4.8) software and stored as moderate resolution tiff files. Images were subsequently cropped and the fluorescent intensity adjusted using ImageJ software.

Numbers of autosomal MLH1 foci in late pachytene cells were manually scored. Only cells characterized by (i) the complete merger of SYCP3 signals from the two homologues, (ii) a full complement of chromosomes, (iii) clear, brightly stained MLH1 foci, and (iv) minimal background fluorescence were scored. We retained only cells with at least one MLH1 focus on each synaptonemal complex, excepting the possibility of one achiasmate bivalent; cells with more than two synaptonemal complexes lacking a MLH1 focus were extremely rare and likely represent staining artifacts. Approximately 20 cells were scored per animal. We were unable to obtain a sufficient number of high quality images for 39 of the 315 F2 animals.

### Genotyping and Data Cleaning

DNA from each F2 animal was extracted from liver tissue using a Wizard Genomic DNA Purification Kit (Promega) following manufacturer's protocols. 295 SNPs distinguishing PWD/PhJ and CAST/EiJ alleles were identified from Phase 4 of the Perlegen mouse resequencing project (Frazer et al. 2007) and genotyped using the Sequenom iPLEX (San Diego, CA) MassARRAY system as previously described [92]. Raw genotype data were cleansed of putative genotype errors and non-Mendelian inheritances as described [80]. A total of 222 high quality SNPs, with an average call rate of 94.2% per SNP, were retained.

### QTL Mapping

A F2 genetic linkage map was constructed using the *est.map* function in the qtl add-on package for R [93]. Recombination fractions were converted to map distances using the Carter-Falconer mapping function [42], [94]. We assumed no genotype error for map construction. Although a few base miscalls might have survived our data cleaning procedure, including a very small number of errors will have a negligible effect on map length estimation.

Multiple QTL mapping was performed using the forward/backward model selection algorithm implemented in the R/qtl command *stepwiseqtl*. Model fitting was performed via extension of Haley-Knott regression [95], with genotype probabilities calculated along a 1 cM grid. Model comparisons were conducted using a penalized LOD score approach with penalties calculated from 1000 permutations of the data [93]. Because biases may be introduced in the stepwise addition of new QTL to the model [93], we repeated the model search multiple times. Each search converged on an identical model of eight QTL and zero epistatic interactions.

## Supporting Information

Figure S1 Relative mRNA transcript abundance for candidate genes within 1.5 LOD units of the X chromosome QTL peak at 33 cM. Expression levels were assayed via real-time quantitative PCR and standardized to levels of β-actin transcript abundance. Expression levels were compared between (A) 4 high versus 4 low mean MLH1 count F2s, (B) inbred CAST versus PWD strains, (C) inbred CASTxPWD F1 males versus a PWDxCAST F1 male, and (D) animals with a CAST X chromosome (*i.e.* the four high mean MLH1 count F2s, inbred CAST, and CASTxPWD F1 males) versus animals with a PWD X chromosome (*i.e.* the four low mean MLH1 count F2s, inbred PWD, and a PWDxCAST F1). ^*^ *P*<0.05.
(PDF)

Table S1 Primer Sequences.
(DOC)

Text S1 Supplementary text.
(DOC)
