# Supplementary figures and images for: Genetic Analysis of Genome-Scale Recombination Rate Evolution in House Mice
Source: PLoS Genet. 2011 Jun 9;7(6):e1002116. doi: 10.1371/journal.pgen.1002116 (PMC3111479; doi:10.1371/journal.pgen.1002116)

**A****High vs. Low F2**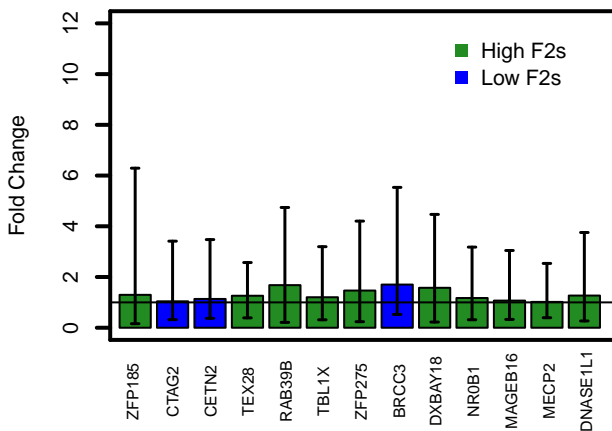**B****PWD vs CAST**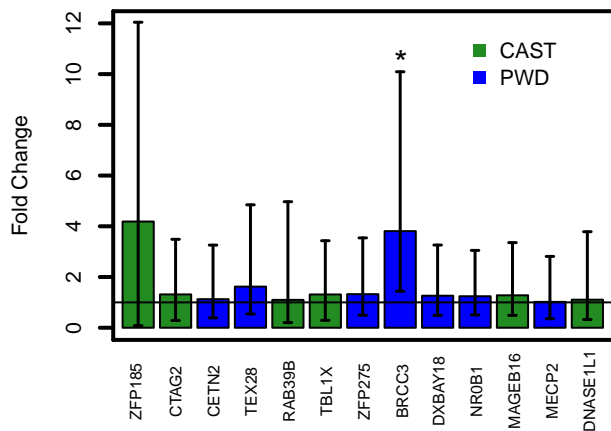**C****CASTxPWD F1 vs PWDxCAST F1**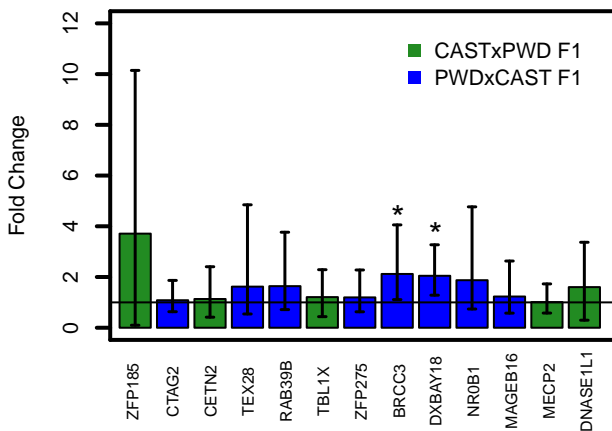**D****CAST X vs PWD X**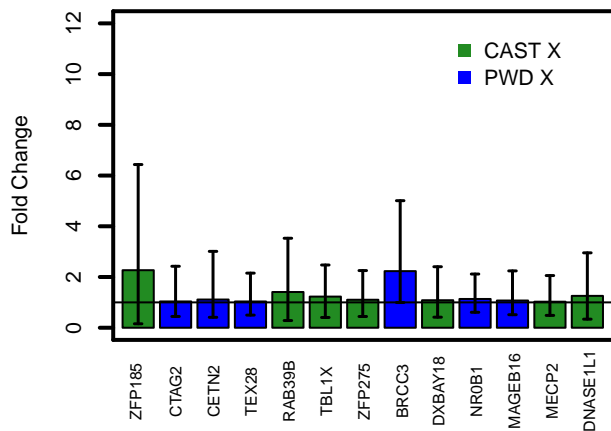

Supplement: Figure S1 — Relative mRNA transcript abundance for candidate genes within 1.5 LOD units of the X chromosome QTL peak at 33 cM. Expression levels were assayed via real-time quantitative PCR and standardized to levels of β-actin transcript abundance. Expression levels were compared between (A) 4 high versus 4 low mean MLH1 count F2s, (B) inbred CAST versus PWD strains, (C) inbred CASTxPWD F1 males versus a PWDxCAST F1 male, and (D) animals with a CAST X chromosome (i.e. the four high mean MLH1 count F2s, inbred CAST, and CASTxPWD F1 males) versus animals with a PWD X chromosome (i.e. the four low mean MLH1 count F2s, inbred PWD, and a PWDxCAST F1). * P<0.05. (PDF) [file pgen.1002116.s001.pdf]
