# Supplementary material for: Genetic Analysis of Genome-Scale Recombination Rate Evolution in House Mice
Source: PLoS Genet. 2011 Jun 9;7(6):e1002116. doi: 10.1371/journal.pgen.1002116 (PMC3111479; doi:10.1371/journal.pgen.1002116)
Supplement: Table S1 — Primer Sequences. (DOC) [file pgen.1002116.s002.doc]

**Table S1.**

| **Gene** | **Forward Primer** | **Reverse Primer** | **Amplicon**  **Size** | **Notes** |
| --- | --- | --- | --- | --- |
| Ldoc | CAAGCCCTGTGCATGGAGAA | GGACAGCTCGGTGGACATAC | 101 | non-specific amplification |
| Actb | GGCTGTATTCCCCTCCATCG | CCAGTTGGTAACAATGCCATGT | 154 |  |
| Nr0b1 | GGGCAGCATCTTATACAGCTTG | CACTCTGGGTACAGTAGGACAG | 116 |  |
| Mageb16 | AATGGGAATCTATGCTGGGATGA | TCACTATTGGCTATTGGCTGGTA | 114 |  |
| Tbl1x | AGCGACGAGGTGAACTTTCTG | GGGACGCCCATCAAATACCG | 219 |  |
| Rab39b | ACCAGTTCCGGCTCATTGTC | GGCTCGATCTCCACCAAACG | 142 |  |
| Brcc3 | AGGTCGTCCCATGAGAGTTG | GGCTTGTATGGATTGGAAGC | 202 |  |
| Dnase1l1 | GTTCCTGATGCACACATAGCA | TCAGTCTGTGGGCATTGAAGG | 112 |  |
| Tex28 | TGCTTTTGCAGAAGATGAAGG | GGCCATTTTCTCTTCAGTGC | 245 |  |
| Mecp2 | TGACTTCACGGTAACTGGGAG | TTTCACCTGAACACCTTCTGATG | 166 |  |
| Naa10 | GGAAGATTGTGGGCTACGTC | ATGCAGGGAGACGTATTTGG | 182 | non-specific amplification |
| Trex2 | ACTGGGCTCCCAAACATGG | CAAGGAACCAGAATCATCCCG | 102 | non-specific amplification |
| Zfp92 | CAAAGCCCAAGGTGACAGTG | CAGCATCACTTGCTTGTAGAGG | 110 | non-specific amplification |
| Zfp275 | GAGGCCCAGGATTTGGTGTC | AGCAGGACTCACAACAGGAAC | 147 |  |
| Ctag2 | CCTAGTGGAGATGCTGCAACA | CTGCTGCTGGATATTTGAGGC | 126 |  |
| Fate1 | TGGGTGCCAAGAGAGCATAG | GTAAAGAGCGCAGCCTCCTTA | 242 | non-specific amplification |
| Cetn2 | TGAAATTGATAAAGAAGGAACAGG | ACCTCACCATCTCCATCTCG | 249 |  |
| Zfp185 | CTGGGAGCAGCTAATTCTGG | GACCTCCGTTTCTGCTCATC | 161 |  |
| DXBay18 | TTTGAGCGCAGGGGTATCC | GGTCGATCACTGAGAAGCACC | 133 |  |
