# Supplementary material for: Genetic Analysis of Genome-Scale Recombination Rate Evolution in House Mice
Source: PLoS Genet. 2011 Jun 9;7(6):e1002116. doi: 10.1371/journal.pgen.1002116 (PMC3111479; doi:10.1371/journal.pgen.1002116)
Supplement: Text S1 — Supplementary text. (DOC) [file pgen.1002116.s003.doc]

**SUPPLEMENTARY TEXT**

*Candidate Gene Identification*

We identified candidate recombination rate modifying genes within 1.5 LOD units of the maximum LOD peak under the X-linked QTL at 33 cM. Specifically, we focused on the subset of genes within this interval that (i) are expressed in the testis and (ii) have known roles in chromatin remodeling, DNA repair, or other processes consistent with a possible effect on recombination. We also included several genes of unknown function that are highly up-regulated in testis. In total, we evaluated 18 genes for expression differences between CAST and PWD alleles (Supplementary Table 1).

*mRNA Extraction and Real-Time qPCR*

Total mRNA was extracted from whole testis tissue using an Aurum Total mRNA mini Kit (BioRad) and converted to cDNA using an iScript^TM^ cDNA Synthesis Kit (BioRad) following manufacturers protocols.

RTqPCR primer pairs and corresponding annealing temperatures are provided in Table S1. Triplicate 20 μL reactions were set up using 1 μL of a 1:2 dilution of cDNA and iQ^TM^ SYBR® Green Supermix (BioRad). Reactions were cycled on a MyiQ^TM^ Single Color Real Time PCR Detection System (BioRad) according to the following program: 3’ at 72° followed by 50 cycles of 10’’ at 95°, 30’’ at primer-specific annealing temperature, 30’’ at 72°. Melt curve analysis was performed on all PCR products to ensure the presence of single amplicons and no primer dimerization. Data from reactions with non-specific amplification and outlier cycle threshold estimates (> 1 cycle compared with duplicate runs) were discarded prior to analysis. Data from 5 candidate genes were completely discarded on the basis of these criteria (Table S1). Cycle threshold values were converted into ΔΔCt values using a multiple linear model framework as described [1], with β-actin expression levels used as an internal standard.

**REFERENCE**

1. Yuan, JS, Reed A, Chen F, and Stewart CN, (2006) Statistical analysis of real-time PCR data. BMC Bioinformatics 7: 85.
